# The molecular effects underlying the pharmacological activities of daphnetin

**DOI:** 10.3389/fphar.2024.1407010

**Published:** 2024-07-01

**Authors:** Zhifeng Wei, Na Wei, Long Su, Sujun Gao

**Affiliations:** ^1^ Department of Hematology, The First Hospital of Jilin University, Changchun, China; ^2^ Department of Obstetrics, The Affiliated Taian City Central Hospital of Qingdao University, Taian, China

**Keywords:** daphnetin, molecular effects, signaling pathways, NLRP3 inflammasome, inflammatory factors

## Abstract

As an increasingly well-known derivative of coumarin, daphnetin (7,8-dithydroxycoumarin) has demonstrated various pharmacological activities, including anti-inflammation, anti-cancer, anti-autoimmune diseases, antibacterial, organ protection, and neuroprotection properties. Various studies have been conducted to explore the action mechanisms and synthetic methods of daphnetin, given its therapeutic potential in clinical. Despite these initial insights, the precise mechanisms underlying the pharmacological activities of daphnetin remain largely unknown. In order to address this knowledge gap, we explore the molecular effects from the perspectives of signaling pathways, NOD-like receptor protein 3 (NLRP3) inflammasome and inflammatory factors; and try to find out how these mechanisms can be utilized to inform new combined therapeutic strategies.

## 1 Introduction

Herbal medicines have been used for thousands of years. Even in recent years, not only in developing countries but also in developed countries, including Europe and North America, it is estimated that more than 50% of the population has used herbal medicinal approaches at least once (Bhoi et al., 2023). Furthermore, herbal medicine has captivated the attention of scientists to probe bioactive compounds derived from natural sources for future drug discovery.

**FIGURE 1:**
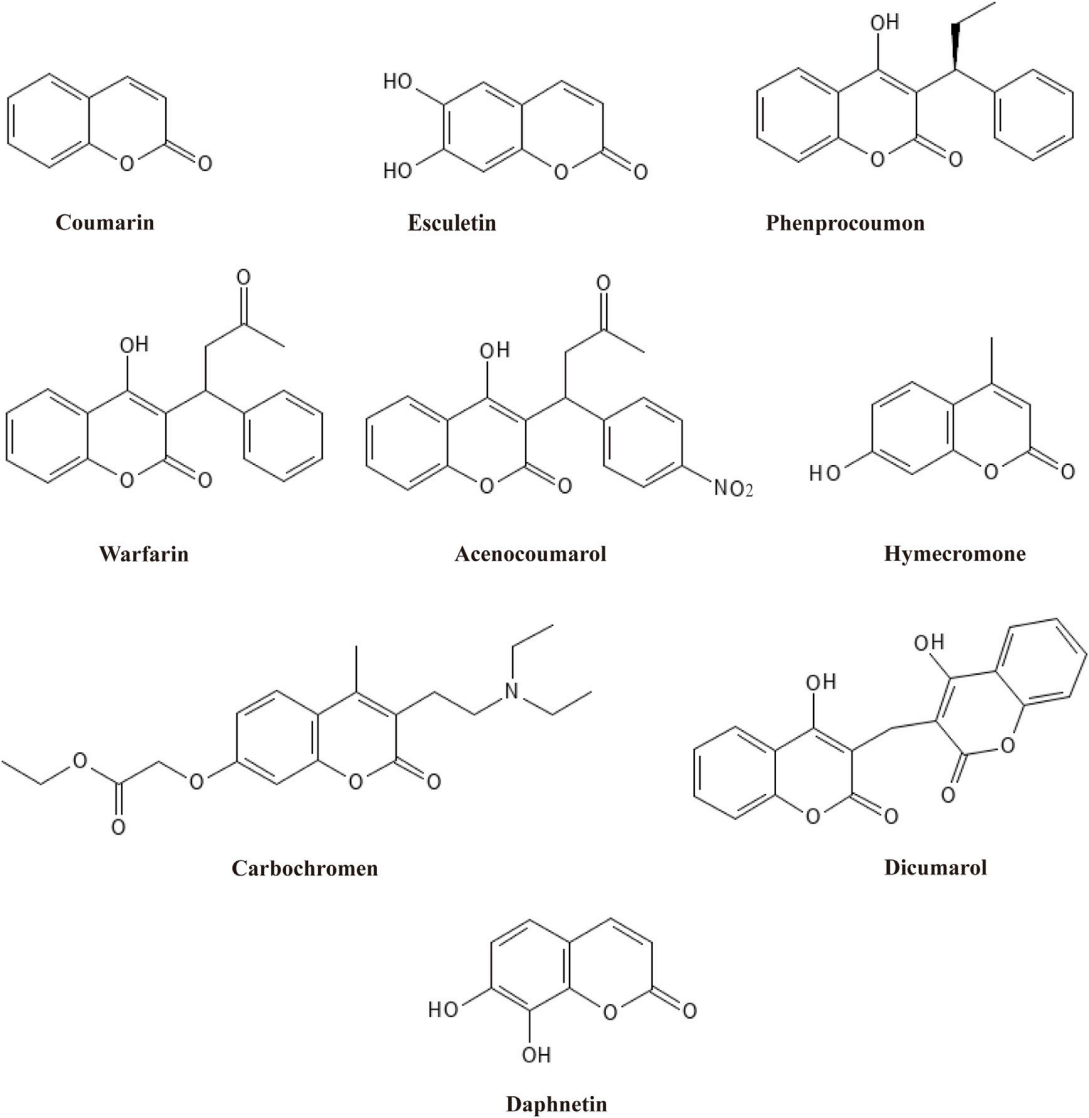
The structures of coumarin-contained drugs widely used and daphnetin.

Among plant active metabolites, coumarins and their derivatives are prominent paradigms and have been used widely. Coumarin, first isolated from tonka beans and melilot flowers by *Vogel* in 1820, is considered the most basic heterocyclic compound with fused phenolic benzene and α-pyrone rings (Ando et al., 2018). As secondary metabolites, coumarins have been found in bacteria, fungi, and about 150 species of plants, where more than 1,300 natural-based coumarins are isolated and identified (Iranshahi et al., 2009). Based on their chemical structure, coumarins are classified into six main types: simple coumarins, furanocoumarins, dihydrofuranocoumarins, phenylcoumarins, pyranocoumarins, and biscoumarins (Hassanein et al., 2020). All six types comprise a coumarin moiety and exert diverse medical functions via their distinct structural characteristics. Therefore, coumarins have been widely used in complementary and alternative medicine owing to their potent and comprehensive pharmacological activities, including anti-inflammatory (Min et al., 2023), antibacterial (Zeng et al., 2023), antiviral (Hwu et al., 2022), antioxidant (Sultana et al., 2022), anti-Alzheimer’s Disease (AD) (Liu W. et al., 2022), and antitumor effects (Ahmed et al., 2022). Based on this, a variety of derivative drugs containing a coumarin moiety have been developed and used in clinics, such as esculetin, phenprocoumon, warfarin, acenocoumarol, hymecromone, carbochromen, dicoumarol, and daphnetin (Figure 1). Structural modification of coumarin can derivate new compounds with potent bioactivities. Of all the derivative drugs, warfarin is the most famous. Warfarin has been widely used as an oral anticoagulant medication for prophylaxis and treatment of venous thrombosis and thromboembolic events (Yaghi et al., 2022).

**FIGURE 2:**
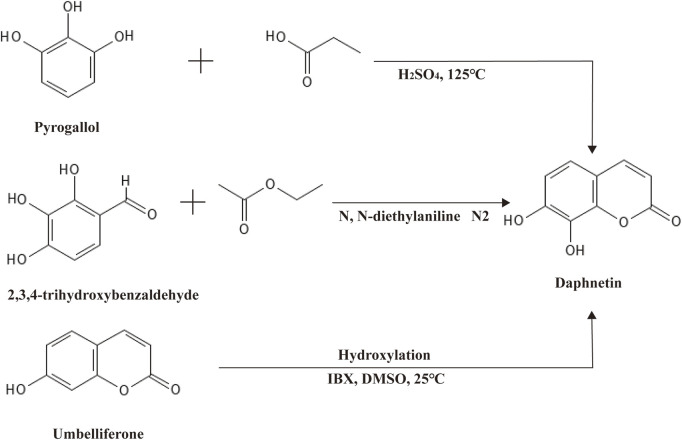
Synthesis of daphnetin via umbelliferone.

Daphnetin, another well-known derivative of coumarin, was first isolated from plants of the *Daphne* genus, hence the name (Wróblewska-Łuczka et al., 2023). Like coumarin, daphnetin is a plant secondary metabolite widely distributed in food and medicinal herbs, especially in Chinese medicinal herbs. As a consequence, daphnetin can be extracted from a variety of natural plants, such as *D. gnidium, D. giraldii, D. mezereum, D. oleoides,* and so on (Han et al., 2020; Khouchlaa et al., 2021). Chemically, daphnetin includes an essential coumarin-like backbone, yet it owns two more hydroxyl groups at C-7 and C-8 compared to coumarin. Thus, daphnetin is also called 7,8-dithydroxycoumarin (Figure 1). Physically, daphnetin exists as an odorless and tasteless powder, dissolving freely in ethanol, methanol and dimethyl-sulfoxide but water slightly (Zhu et al., 2010). As a natural product, daphnetin was mainly extracted from plants at first, which limited its large-scale utilization. Though there is no report suggesting that coumarin can be synthesized via coumarin, daphnetin can be synthesized from pyrogallol, 2,3,4-trihydroxybenzaldehyde, and umbelliferone (7-hydroxycoumarin) as illustrated in Figure 2 (Bizzarri et al., 2017; Wang et al., 2017; Pardo-Castaño et al., 2019). When pyrogallol and propionic acid are heated at 125 C, daphnetin can be synthesized under the catalysis of concentrated sulfuric acid (Pardo-Castaño et al., 2019). Daphnetin is synthesized when 2,3,4-trihydroxybenzaldehyde and ethyl acetate are chemical reaction substrates in the presence of N, N-diethylaniline under a nitrogen atmosphere (Wang et al., 2017).

**FIGURE 3:**
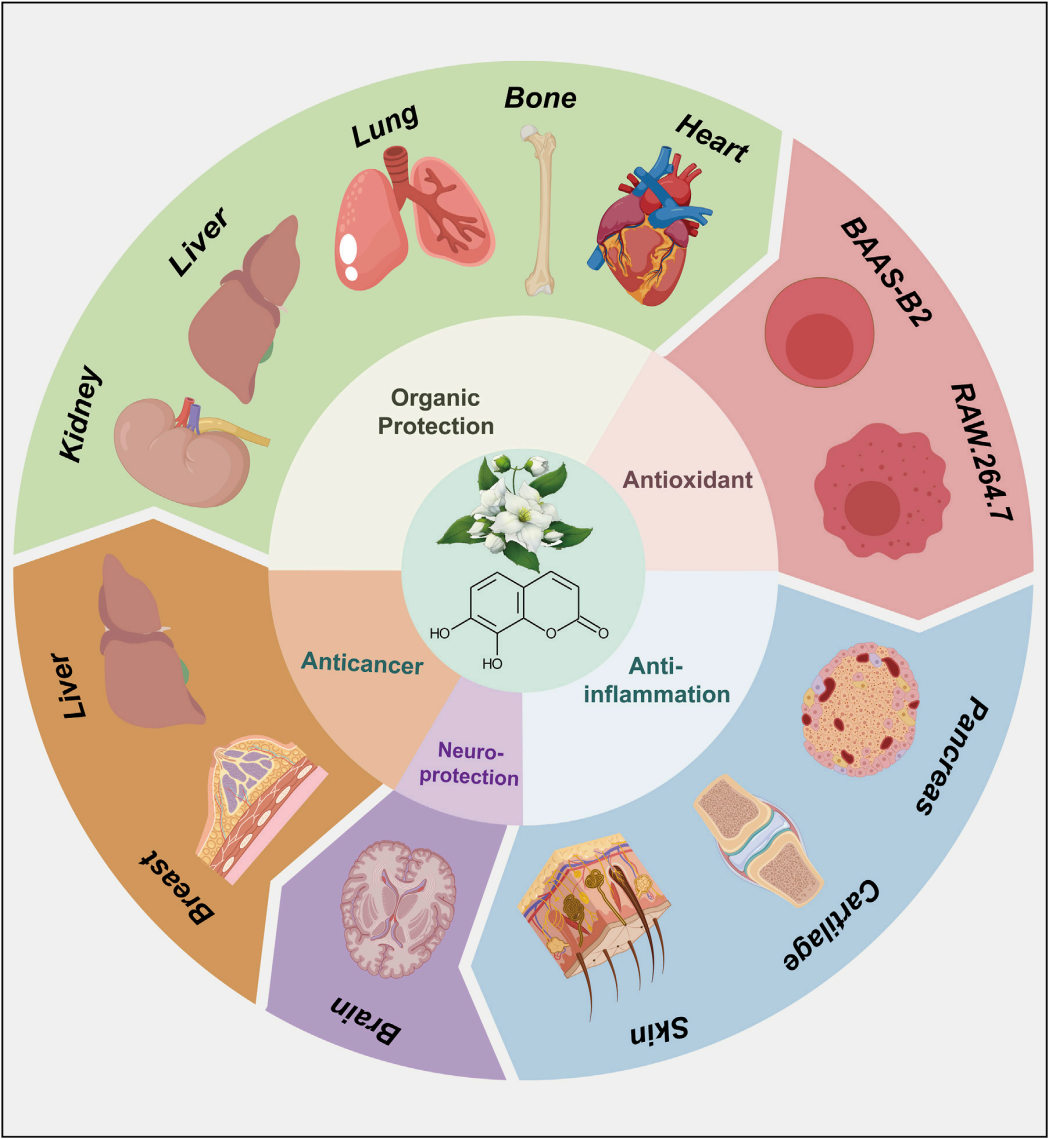
The pharmacological activities of daphnetin.

**FIGURE 4:**
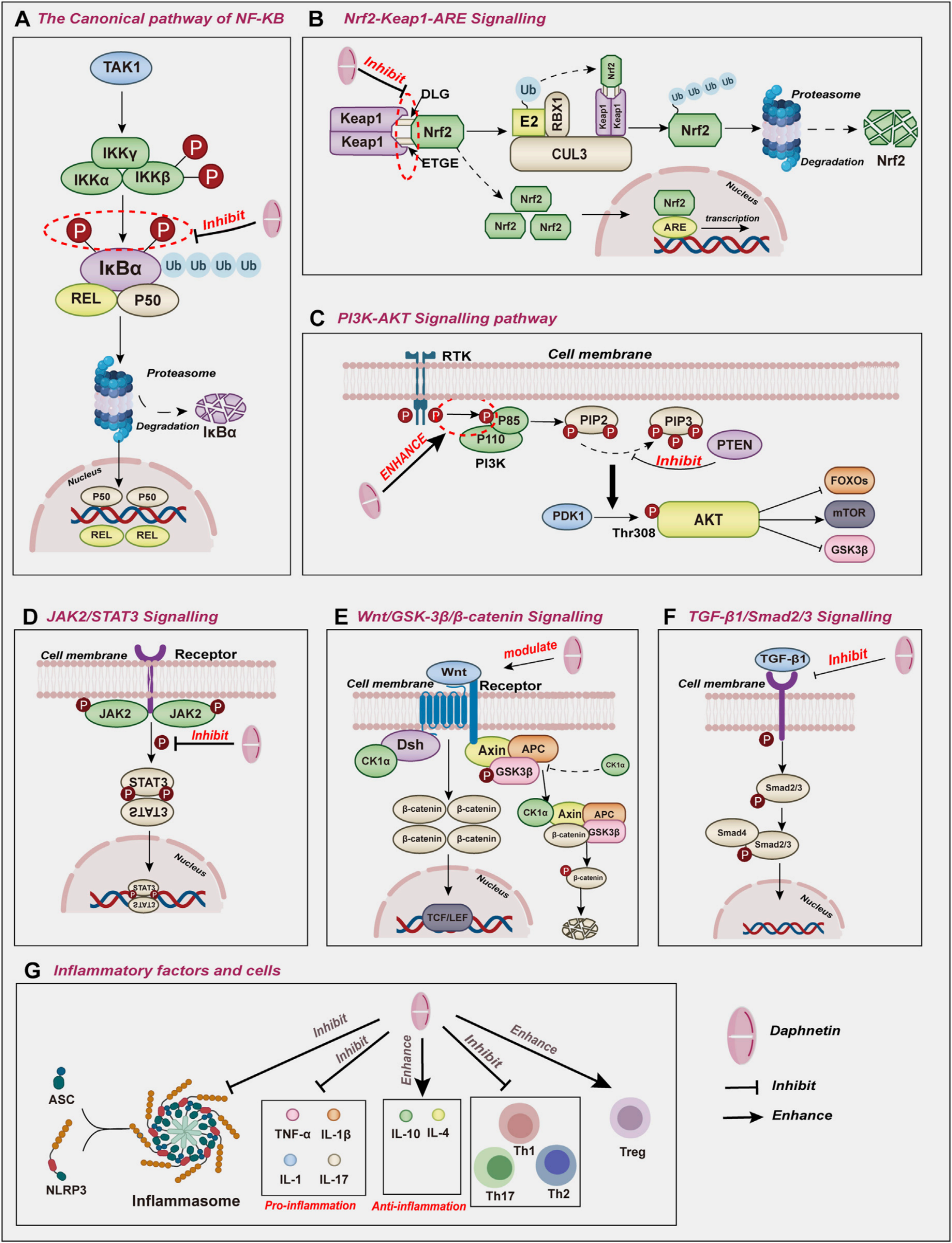
The molecular effects underlying the pharmacological activities of daphnetin include **(A)** NF-κB, **(B)** Nrf2, **(C)** PI3K/AKT, **(D)** JAK2/STAT3, **(E)** Wnt/GSK-3β/βcatenin, **(F)** TGF-β1/Smad2/3signaling pathways and **(G)** inflammatory factors and cells.

As a representative derivative of coumarins, the biological activities of daphnetin have drawn much attention among scientists and have been the subject of extensive research (Wang et al., 2020b; Boulebd and Amine Khodja, 2021; Pei et al., 2021). Since the 1980s, daphnetin has been an adjunctive therapy for cardiovascular diseases (Gao et al., 2008). Additionally, daphnetin has been increasingly identified as an essential compound of the Zushima tablet, a traditional Chinese medicine preparation used to treat rheumatoid arthritis (Han et al., 2020). Recently, more pharmacological activities of daphnetin have been reported, including anti-inflammation, anti-cancer, anti-autoimmune diseases, and neuroprotection properties (Figure 3) (Finn et al., 2004; Shu et al., 2014; Lv et al., 2018; Zhi et al., 2019; Yan et al., 2022; Zhang et al., 2022). Although the bioactivities and therapeutic potentials of daphnetin have been well documented (Hang et al., 2022; Javed et al., 2022), the elaborate molecular mechanisms associated with its functions remain largely unknown. Here, we appreciate more attention to the molecular effects underlying the pharmacological activities of daphnetin. We intend to explore how daphnetin performs its pharmacological effects via distinct signaling pathways, NLRP3 inflammasome, and inflammatory factors (Figure 4; Table 1) to provide insights for developing new therapeutic strategies for relevant diseases.

**TABLE 1:** The pharmacological activities and underlying mechanisms of daphnetin.

Molecular effects	Targets	Pharmacological effects	Models/Methods	Dose	Research	Results stage	Ref.
Pathways	NF-κB pathway	Anti-inflammation	Rat acute pancreatitis model	4 mg/kg	Acute pancreatic injury is alleviated	Animal experiment	Liu et al. (2016)
		Chondroprotective and antiarthritic properties	Rabbit osteoarthritis model	12, 24 and 48 ng/mL	Chondrocytes and articular cartilage are protected	Animal experiment	Zhang et al. (2020a)
		Protective properties	Endotoxin-induced pulmonary injury model	5 and 10 mg/kg	Pulmonary injury is suppressed	Animal experiment	Yu et al. (2014)
		Hepatoprotective and anti-inflammation properties	LPS/GalN-induced mice ALF model	20, 40, and 80 mg/kg	ALF and its complications are suppressed	Animal experiment	Lv et al. (2018)
		Neuroprotective properties	EAE mice model for MS	8 mg/kg	Clinical symptoms of EAE are alleviated	Animal experiment	Wang et al. (2016)
		Anti-inflammation properties	NZB/W F1 SLE model	5 mg/kg	Survival rate and damage of SLE are improved	Animal experiment	Li et al. (2017)
		Anti-cancer properties	DMBA-induced breast carcinoma model	20, 40, 80 mg/kg	Breast carcinogenesis is inhibited	Animal experiment	Kumar et al. (2016)
		Anti-cancer and hepatoprotective properties	Chemically induced hepatocellular carcinoma	10, 20, and 30 mg/kg	Hepatocellular carcinoma incidence and symptoms are suppressed	Animal experiment	Li et al. (2022)
		Anti-cancer properties	HMGB1 induced A549 cell EMT model	10–80 mg/kg	Epithelial-mesenchymal transition of A549 cells is inhibited	Cell experiment	Gong et al. (2023)
	Nrf2 pathway	Antioxidant properties	t-BHP-induced RAW 264.7 cells dysfunction	2.5, 5, and 10 μg/mL	RAW 264.7 cells are protected against t-BHP-induced oxidative damage	Cell experiment	Lv et al. (2017)
		Antioxidant properties	NaAsO2-induced Beas-2B-cells cytotoxicity	2.5, 5, and 10 μg/mL	Beas-2B-cells are protected from oxidative stress and cytotoxicity	Cell experiment	Lv et al. (2019)
		Renoprotection properties	GM-induced renal injury mice model	40 mg/kg	GM-induced nephrotoxicity is inhibited	Animal experiment	Fan et al. (2021a)
		Renoprotection properties	Cisplatin-induced nephrotoxicity mice model	40 mg/kg	Cisplatin-induced Nephrotoxicity is reversed	Animal experiment	Zhang et al. (2018)
		Hepatoprotective properties	APAP or t-BHP-induced ALF mice model	40 and 80 mg/kg	Hepatotoxicity is alleviated	Animal experiment	Lv et al. (2020)
		Hepatoprotective properties	CCl4-induced hepatotoxicity rat model	4.5 mg/kg	Hepatotoxicity related to oxidative stress is ameliorated	Animal experiment	Mohamed et al. (2014)
	PI3K/AKT	Immunoregulatory properties	Cells co-culture	10 μM	The activation of NK cells is enhanced	Cell experiment	Yao et al. (2021)
		Neuroprotective properties	Alzheimer’s disease mice model	2, 4, and 8 mg/kg	Memory impairment is mitigated	Animal experiment	Yan et al. (2022)
		Chondroprotective and antiarthritic properties	CIA rat model	0–60 μg/mL	The proliferation of CIA-FLS and autophagy is inhibited	Animal experiment	Deng et al. (2020)
		Anti-cancer properties	A2780 xenograft mice model	30 mg/kg	Ovarian cancer is inhibited	Animal experiment	Fan et al. (2021b)
	JAK2/STAT3	Anti-inflammation and antioxidant properties	DSS-induced UC mouse model	16 mg/kg	Colitis and intestinal structure in DSS-induced mice are attenuated	Animal experiment	He et al. (2023)
		Anti-inflammation properties	PALI mice model	2–4 mg/kg	The severity of pancreatic and lung injury is reduced	Animal experiment	Yang et al. (2021)
		Anti-inflammation properties	LPS induced Raw264.7 cells inflammation	5–20 μM	LPS-induced ROS production is suppressed	Cell experiment	Shen et al. (2017)
	Wnt/GSK-3β/β-catenin	Osteoprotective properties	glucocorticoid-induced osteoporosis rat model	1 and 4 mg/kg	The symptoms and biochemical markers of GIOP are ameliorated	Animal experiment	Wang et al. (2020a)
		Anti-cancer and hepatoprotective properties	Xenograft HCC cells mice models	25 and 50 mg/kg	viability and tumorigenesis of HCC cells are inhibited	Animal experiment	Liu et al. (2022a)
	TGF-β1/Smad2/3	Antioxidant and cardioprotective properties	The transverse aortic constriction mice model	10 and 20 mg/kg	Ischemia/reperfusion injury and cardiac function are improved	Animal experiment	Syed et al. (2022)
	Autophagy	Antibacterial and anti-inflammation properties	S. aureus-induced model pneumonia	10 mg/kg	Inflammatory responses are reduced and bacterial clearance is augmented	Animal experiment	Zhang et al. (2019)
Inflammasome	NLRP3	Anti-inflammation and hepatoprotective properties	LPS/GalN-induced mice ALF model	20, 40, and 80 mg/kg	ALF and its complications are suppressed	Animal experiment	Lv et al. (2018)
Inflammatory reaction	Inflammatory factors	Anti-inflammation and neuroprotective properties	Neuropathic pain rat model	12.5 μg/rat	Neuropathic pain is ameliorated	Animal experiment	Zhang et al. (2023)
		Anti-inflammation and neuroprotective properties	EAE mice model	2 and 8 mg/kg	The symptoms of EAE are alleviated	Animal experiment	Soltanmohammadi et al. (2022)
		Anti-cancer and hepatoprotective properties	Chemically induced HCC rat model	10, 20, and 30 mg/kg	Expansion of HCC is ameliorated	Animal experiment	Li et al. (2022)
	Th cells	Chondroprotective and antiarthritic properties	CIA rat model	1 and 4 mg/kg	The symptoms of CIA are alleviated	Animal experiment	Tu et al. (2012)
		Immunoregulatory and antiarthritic properties	CIA rat model	1 and 4 mg/kg	The severity of the arthritis is alleviated	Animal experiment	Yao et al. (2011)
		Maintaining the balance of Th cells	PBMC from patients with URPL loss assay	20 and 40 μg/mL	Th17 and Treg cells in URPL are balanced	Cell experiment	Zhang et al. (2020c)
		Anti-inflammation and neuroprotective properties	EAE mice model for MS	8 mg/kg	Clinical symptoms of EAE are alleviated	Animal experiment	Wang et al. (2016)

ALF, acute liver failure; EAE, experimental autoimmune encephalomyelitis; MS, multiple sclerosis; SLE, systemic lupus erythematosus; DMBA, 7,12-dimethylbenz(a)anthracene; t-BHP, tert-butyl hydroperoxide; GM, gentamicin; APAP, acetaminophen; CIA, collagen-induced arthritis; FLS, fibroblast-like synoviocytes; UC, ulcerative colitis; PALI, SAP-associated acute lung injury.

To determine whether elaborate evidence elucidates the molecular effects underlying the pharmacological activities of daphnetin, we conducted a comprehensive search across multiple reputable databases, including MEDLINE, EMBASE, Web of Science, and Google Scholar, to identify relevant articles published. The search strategy used a combination of MeSH terms pertaining to signaling pathways and daphnetin (7,8-dithydroxycoumarin) and Boolean operators to retrieve articles. The inclusion criteria to guide the selection process was as follows: well-controlled intervention studies in cell lines and animal models detecting the changes of signaling pathways in the presence of daphnetin. Studies that did not assign a control group and irrelevant literature were excluded. Two independent investigators conducted an initial screening of the articles based on the inclusion criteria above. Articles that met the predefined inclusion criteria were selected for a full-text assessment. Any discrepancies between investigators were resolved by discussion until a consensus was reached. Subsequently, based on this point, we proposed the possible signaling pathways, inflammation cells and factors linked to the pharmacological activities of daphnetin and promising combined therapeutic strategies.

## 2 Effects of daphnetin on signaling pathways

### 2.1 Effects of daphnetin on NF-κB signaling pathway

Nuclear Factor-kappa B (NF-κB), an essential transcription factor, has been reported to exert an increasingly fundamental role in regulating inflammatory and immune responses (Zhang et al., 2017; Yu et al., 2020; Fang et al., 2023). NF-κB family consists of five prominent inducible members: RELA (p65), RELB, c-REL, NF-κB1 (p50), and NF-κB2 (p52). These proteins interact with each other to form distinct homodimers and exert their physiological functions (Ghosh et al., 1998). NF-κB proteins usually are kept in inactive, as bound to the inhibitor of κB (IκB) family. Various inflammatory stimuli can trigger the phosphorylation of IκB, which leads to its degradation and the release of the active NF-κB dimer (Esposito et al., 2016).

NF-κB can be activated through the canonical pathway, which responds to various external stimuli involved in inflammation and immune responses such as cytokines, pathogens, and stress (Lawrence, 2009; Sun, 2017). NF-κB1, RELA, and c-REL are activated and translocated to the nucleus to mediate downstream gene transcription in the canonical pathway (Figure 4A).

Recently, mounting evidence shows that daphnetin possesses multiple bioactivities via regulating the NF-κB signaling pathway in various exogenous inflammatory animal models. Western blot analysis reveals that the NF-κB signaling pathway is over-activated in a rat severe acute pancreatitis model induced by sodium taurocholate. Pretreatment of daphnetin at 4 mg/kg significantly blocks the TLR4/NF-κB signaling pathway by inhibiting the phosphorylation of IκBα and the expression of TLR4, thereby attenuating pancreatic injury in rat severe acute pancreatitis (SAP) model compared to the control group (Liu et al., 2016). Similarly, in a rabbit model of osteoarthritis called the Hulth-Telhag model, Zhang et al. found that daphnetin exerted a fundamentally chondroprotective role in Hulth-Telhag rabbit chondrocytes (Rogart et al., 1999). Furthermore, *in vitro* assays suggested that daphnetin (12, 24 and 48 ng/mL) markedly suppressed the expression of matrix metalloproteinases MMP-3, MMP-9 and MMP-13 in synovial cells, which is partially due to the inhibition of NF-κB signaling pathways and subsequent downregulation of IL-1β, IL-6, IL-12 (Zhang X. et al., 2020). Daphnetin at 5 and 10 mg/kg also exerts anti-inflammatory and protective properties in a mouse endotoxin-induced lung injury (EILI) model, a severe inflammatory condition caused by bacterial toxins (Yu et al., 2014). Subsequently, both *in vitro* and *in vivo* experiments indicated that daphnetin decreased the levels of inflammatory cytokines, reactive oxygen species, and apoptotic markers in EILI by modulating the NF-κB signaling pathways in a dose-dependent manner at a range from 10 to 160 μM, which partly explains the role of daphnetin (Yu et al., 2014). Likewise, in a mouse acute liver failure (ALF) model induced via lipopolysaccharide (LPS)/D-galactosamine (GalN), the NF-κB signaling pathway is involved in the process of inflammation, mediating the occurrence and progression of ALF. Compared with the LPS/GalN-challenged group, daphnetin at 20, 40, and 80 mg/kg effectively and dose-dependently inhibited JNK, ERK, and P38, blocking the phosphorylation and degradation of IκBα. Therefore, daphnetin decreased the translocation of NF-κB (p65), significantly induced autophagy activation and finally prolonged the survival of the treatment group, suggesting that the anti-inflammation property of daphnetin is partly attributed to the inhibition of the NF-κB signaling pathway activation (Lv et al., 2018).

By modulating the NF-κB signaling pathway, daphnetin can also relieve several auto-immune inflammation diseases. Given that autoreactive T-cell has been implicated in the pathogenesis of a variety of autoimmune diseases (Rosetti et al., 2022), researchers have focused on the inhibitory effects of daphnetin(0–64 mg/mL) at 48 h and 24 h on concanavalin A (ConA) induced T-cell proliferation and found that daphnetin significantly suppressed splenocyte proliferation and cell cycle progression by blocking the G0/G1 transition and the NF-κB pathway activation in mouse T-cell, finally mediating immunosuppressive activity on T-cell (Song et al., 2014). As one of the most common chronic disabling neurological diseases, multiple sclerosis (MS) mainly affects young adults and is closely related to aging (Zhang Y. et al., 2020; Graves et al., 2023). In a mice experimental autoimmune encephalomyelitis (EAE) animal model for MS, constant administration of daphnetin(8 mg/kg) for 28 days markedly alleviated the clinical symptoms and demyelination of the mouse by repressing Th1 and Th17 cell responses. Mechanistically, daphnetin repressed the phenotype and function of dendritic cells via modulating NF-κB signaling pathways (Wang et al., 2016). Recently, daphnetin has been used to intervene in systemic lupus erythematosus (SLE), a systemic autoimmune disease with multiple pathogenic factors and organ involvement (Gatto et al., 2013). In an SLE NZB/WF 1 mouse model, administration of daphnetin (at 5 mg/kg) once a day for 12 weeks reduced the organ damage caused by SLE, lowered the serum autoantibody production, and increased the survival rate of mice with SLE via suppressing RELA and NF-κB signaling pathways (Gatto et al., 2013). Furthermore, phosphorylation and degradation of IκBα, the indicators of NF-κB activation, are prevented by daphnetin (Li et al., 2017).

Moreover, daphnetin has been identified to prevent against cancer via modulating NF-κB signaling pathways. In Sprague-Dawley rats with mammary carcinogenesis induced by 7,12-dimethylbenz(a)anthracene (DMBA), constant daphnetin treatment for 28 days at 20, 40, 80 mg/kg dose-dependently corrected these inflammatory changes as well as enhanced the antioxidative protection in these cancer-bearing animals by hindering the expression and nuclear translocation of NF-κB (Kumar et al., 2016). Still, administration of daphnetin at 10, 20, and 30 mg/kg for 16 weeks was verified to ameliorate the invasion of chemically induced hepatocellular carcinoma via reduction of inflammation and oxidative stress in a concentration-dependent manner (Li et al., 2022). Daphnetin suggestively suppressed the tumor incidence and the weight of the liver and spleen in a dose-dependent manner compared with the untreated group. The mechanism of anti-cancer in daphnetin involved the suppression of inflammatory responses via the NF-κB signaling pathway (Li et al., 2022). Furthermore, daphnetin affected the epithelial-mesenchymal transition process in human lung adenocarcinoma cells. Gong et al. (2023) found that daphnetin(10–80 mg/kg) inhibited the proliferation and migration of human lung adenocarcinoma epithelial A549 cells through regulating the NF-κB signaling pathway (Gong et al., 2023).

Consequently, daphnetin exerts its bioactivity via modulating NF-κB pathways, including anti-inflammation and anti-cancer effects.

### 2.2 Effects of daphnetin on Nrf2 signaling

Nuclear factor erythroid 2-related factor 2 (Nrf2) is known as a pivotal transcription factor that belongs to the Cap’n’Collar (CNC) family of conserved basic leucine zipper (bZIP) transcription factors (Moi et al., 1994). Structurally, Nrf2 is composed of seven conserved Nrf2-ECH homology domains (Neh1–7) with different functions to regulate Nrf 2 transcriptional activity (Hayes and Dinkova-Kostova, 2014). Of all Nehs, Neh1 allows the recognition of antioxidant response elements (ARE) to activate gene transcription. Under normal physiological homeostasis, Neh2 is bound to Kelch-like-ECH-associated protein 1 (Keap1), which stimulates CULLIN3(CUL3) E3 ubiquitin ligase and leads to proteasomal degradation of Nrf2 via the proteasome system. This degradation mechanism maintains Nrf2 dynamic equilibrium at the protein level, ensuring only a tiny fraction of Nrf2 reaches the nucleus to regulate the basal expression of target genes (Torrente and DeNicola, 2022). Once stress occurs, inhibition of Nrf2 directed by Keap1 is abrogated, resulting in Nrf2 stabilization (Dayalan Naidu and Dinkova-Kostova, 2020). Consequently, Nrf2 can liberate and translocate into the nucleus to interact with AREs, activating their transcription and antioxidant characteristics (Figure 4B) (Itoh et al., 1997; Kobayashi et al., 2016). Nrf2 has emerged as a pivotal player in various cellular processes maintaining cell homeostasis (Shaw and Chattopadhyay, 2020). By upregulating the Nrf2 pathway, daphnetin can alter the expression levels of Bcl-2, Bax, and caspase 3, critical apoptosis regulators (programmed cell death) in cells exposed to oxidative stress or injury (Liang et al., 2010).

By modulating the Nrf2 signaling pathway, daphnetin protects cells against oxidative damage and mitochondrial dysfunction. The organic peroxide tert-butyl hydroperoxide (t-BHP), acting as a cellular toxin, promotes oxidative stress and leads to various types of cell damage. In a cell apoptosis model induced by t-BHP, daphnetin (2.5, 5, and 10 μg/mL) significantly guarded RAW 264.7 cells against t-BHP-induced cytotoxicity and cell apoptosis in a concentration-dependent manner (Lv et al., 2017). Furthermore, daphnetin decreased t-BHP-induced ROS generation and inhibited the expression of cytochrome c in RAW 264.7 cell cytoplasm and mitochondria, which preserved the mitochondrial physiological function (Lv et al., 2017). Mechanically, daphnetin activated the Keap1-Nrf2/ARE signaling pathway to exert antioxidant effects and modulate the expression of numerous antioxidant enzymes, including GCLC, GCLM, and HO-1 (de Oliveira et al., 2016). However, daphnetin-mediated cell viability, ROS blockade, and the expression of antioxidative enzymes was almost abolished in Nrf2 knockout RAW 264.7 cells, which verified that the role of daphnetin in preventing mitochondrial dysfunction was largely dependent on the upregulation of the Nrf2 pathway (Niture et al., 2014; Lv et al., 2017).

Similarly, daphnetin treatment significantly alleviated the cellular toxic effects of arsenic on human lung epithelial cells (Lv et al., 2019). As a natural toxicant, arsenic is demonstrated to induce acute and chronic toxicity in lung tissue, including tissue injury and cell apoptosis (Putila and Guo, 2011; Wang et al., 2023). Further research showed that daphnetin (2.5, 5 and 10 μg/mL) enhanced Nrf2 activation and translocation and increased Keap1 degradation in Beas-2B cells, which activated genes downstream of Nrf2 in Beas-2B cells to protect Beas-2B cells from arsenic-induced oxidative stress and cytotoxicity (Lv et al., 2019). Significantly, pretreatment with daphnetin reversed the decrease in the anti-apoptotic factor Bcl-2 induced by arsenic and reduced the increase in the pro-apoptotic factor Bcl-2-associated X protein (Bax), which was considerably attenuated when Nrf2 was depleted *in vitro* (Lv et al., 2019).

Not only cellular protection, but also organic preservation is remarkably mediated by daphnetin. Through enhancing the Nrf2 antioxidant signaling pathway, daphnetin inhibits inflammatory and oxidative responses, protects against organic injury and toxicity, and sustains the physiological function and homeostasis of organs (Mohamed et al., 2014; Zhang et al., 2018; Lv et al., 2020; Fan et al., 2021a).

Though known as a potent antimicrobial agent against Gram-negative infections, gentamicin (GM) has limited clinical applicability due to its nephrotoxic effects (Lopez-Novoa et al., 2011; Lee et al., 2012). In a GM-induced renal injury mice model, daphnetin at 40 mg/kg is demonstrated to ameliorate GM-induced kidney dysfunction and cell damage in mice. Pretreatment with daphnetin significantly reduced the level of BUN and creatinine and improved renal appearance and histopathological evaluation in mice impaired by GM (Fan et al., 2021a). Mechanism research showed that daphnetin activated the Nrf2/ARE pathway in a dose-dependent manner, attenuated oxidative stress and inflammation, and protected against tubular cell apoptosis induced by GM (Fan et al., 2021a). Meanwhile, daphnetin (40 mg/kg) markedly ameliorated nephrotoxicity and renal dysfunction induced by chemotherapeutic cisplatin via modulating the Nrf2 pathway (Zhang et al., 2018). Daphnetin lessened both cisplatin-induced kidney biochemical parameters disorder and histopathological changes. Subsequent evidence proved that daphnetin improved the kidney’s oxidative stress and inflammatory reaction, reversed the nephrotoxicity caused by cisplatin, and sustained renal normal physiological function (Zhang et al., 2018).

Based on the remarkably protective effects of daphnetin in t-BHP-induced mitochondrial dysfunction, Lv et al. (2020) verified that daphnetin (40 or 80 mg/kg) alleviated t-BHP and acetaminophen (APAP)-induced hepatotoxicity through altering Nrf2 pathway activation. Moreover, the pharmacological effect was improved with the mounting concentration. It is reported that daphnetin attenuated t-BHP-triggered hepatotoxicity as well as mitochondrial dysfunction in HepG2 cells, protected against APAP-induced acute liver failure in mice, and prolonged the survival of mice treated by APAP. The hepatoprotective mechanism of daphnetin against APAP relies on the regulation of the Nrf2 signaling pathway, and these beneficial effects were eliminated in Nrf2-deficient mice. Additionally, daphnetin suppressed JNK and ASK1 phosphorylation, Txnip and NLRP3 expression, and caspase-3 cleavage in WT mice, which were related to oxidative phosphorylation, inflammation, and apoptosis (Cao et al., 2017; Woolbright and Jaeschke, 2017; Lv et al., 2020).

Another previous research suggested that daphnetin administrated for 4 weeks at 4.5 mg/kg effectively protected the liver from CCL4-induced damage, possibly through its antioxidant and anti-inflammatory effects (Mohamed et al., 2014). Daphnetin restored near control levels of the hepatic enzymes ALT and AST and markedly improved the histopathology of the liver in CCL4-treated mice, indicating its improvement in liver function. Moreover, daphnetin reduced the levels of oxidative stress marker malondialdehyde in the liver tissues of CCL4-treated mice, indicating its ability to suppress inflammatory responses. mRNA analysis revealed that the expression of HO-1, which was dependent on the Nrf2 pathway, was induced. Consequently, daphnetin facilitated Nrf2 nuclear translocation to confer hepatoprotection against oxidative injury (Mohamed et al., 2014).

Accordingly, by regulating the Nrf2 pathways, daphnetin mediates antioxidant damage and mitochondrial maintenance at cellular and organic levels.

### 2.3 Effects of daphnetin on PI3K/AKT signaling

The Phosphatidylinositol 3-kinase (PI3K)/Protein Kinase B (PKB, also named AKT) signaling pathway, is renowned for its pivotal role in regulating various cellular processes, including proliferation, differentiation, and apoptosis (Arcaro and Guerreiro, 2007). Among three PI3Ks (Vanhaesebroeck et al., 2010), Class IA PI3K, a heterodimeric protein, comprises a catalytic (p110) and a regulatory subunit(p85) (Vidal et al., 2022). In normal physiological conditions, catalytic subunits are bonded and inhibited by regulatory proteins. The regulatory proteins bring the catalytic subunits in contact with their lipid substrates at the membranes on cellular activation (Bilanges et al., 2019; Vidal et al., 2022). AKT serine/threonine kinase family plays pivotal roles as key downstream effector molecules in the PI3K signaling pathway (Toulany et al., 2017). When extracellular signals are detected, PI3K is recruited to the plasma membrane and subsequently activated by either receptor tyrosine kinases or G-protein coupled receptors, initiating the conversion of PIP2 into PIP3 (Vanhaesebroeck et al., 2010). Subsequently, AKT and phosphoinositide-dependent kinase 1 (PDK1) are recruited to the inner surface of the plasma membrane. Once at the membrane, PDK1 phosphorylates AKT at Thr308 to initiate AKT activation (Figure 4C) (Toulany et al., 2017).

Subsequent regulatory effects of activated AKT on cellular biological processes are mediated by various downstream target proteins. AKT regulates downstream target proteins through a phosphorylation cascade, including FOXO, mTOR, and GSK3b, to control cell survival, growth, and proliferation (He et al., 2021). Given the essential function of PI3K/Akt signaling including cell proliferation, survival, metabolism and motility, the promising ability of daphnetin to selectively modulate PI3K signaling has garnered increasing attention for its potential development.

Recent studies have identified daphnetin as a natural compound that effectively activates NK cell effector functions. Further research revealed that daphnetin at 10 μM directly improved the cytotoxicity of NK cells and promoted IFN-γ production in the presence of IL-12 (Yao et al., 2021). Subsequent RNA-sequencing analyses demonstrated that the mechanisms of daphnetin in regulating NK cells are dependent on the PI3K-Akt signaling pathway, which is further confirmed by the impact of PI3K-Akt and mTOR inhibitors (such as LY294002 and rapamycin) on daphnetin-mediated NK cell activation (Yao et al., 2021).

Daphnetin (2, 4, and 8 mg/kg) is reported to exert neuroprotective action in Alzheimer’s disease (AD) model mice (Yan et al., 2022). As a progressive neurodegenerative disorder with genetic complexity, AD is clinically characterized by the dysfunction of memory and cognition (Author Anonymous, 2023). Neuropathological features of AD include neurofibrillary tangles, neuroinflammation, and β-amyloid accumulation, which significantly contribute to AD development due to their impact on synaptic function (Author Anonymous, 2023; Jorfi et al., 2023). In AD-linked transgenic model mice, daphnetin alleviated cognitive impairment by reducing β-amyloid deposition compared to the control group. Moreover, daphnetin promotes the dendrite branch density and increases synaptic protein generation via activating PI3K-Akt signaling (Yan et al., 2022). This finding was corroborated by the use of the PI3K inhibitor LY294002, which reversed daphnetin-induced neuroprotective effects.

By targeting the PI3K/AKT/mTOR signaling pathway, Daphnetin(0–60 μg/mL) can inhibit autophagy and relieve inflammation in fibroblast-like synoviocytes (FLS) in rats with collagen-induced arthritis (CIA) induced by TNF-α. Likewise, the pharmacological inhibitive effects increased with the increasing concentration (Deng et al., 2020). In this disease, the PI3K-Akt signaling pathway significantly impacts the migration of FLS and the inhibition of cartilage formation (Su et al., 2019; Weng et al., 2023). Compared to the disease model group, daphnetin reduces the phosphorylation of AKT and mTOR by inhibiting the mRNA expression of AKT and increasing the mRNA expression of the PI3K negative regulatory gene PTEN. Subsequently, PI3K/AKT signaling downstream effector mTOR and BAD, which govern autophagy negatively and apoptosis respectively, are significantly suppressed. Consequently, daphnetin may be a potential therapeutic approach in treating rheumatoid arthritis (Deng et al., 2020).

In addition, daphnetin is believed to possess antitumor potential (Kumar et al., 2018; Deng et al., 2020), and exhibit potent antitumor effects in ovarian cancer by inducing ROS-dependent apoptosis, which relies on the Akt/mTOR pathway (Fan et al., 2021b). Daphnetin inhibited ovarian cancer proliferation and promoted cell apoptosis *in vivo* at 30 mg/kg and *in vitro* at 0, 5, 10, 20, and 40 μg/mL in three different cell types, which was mediated via the production of ROS. Moreover, daphnetin treatment accumulated the level of LC3B-II, autophagic vacuoles, and autophagic flux in ovarian cancer, suggesting that cytoprotective autophagy was activated. Notably, once combined with autophagy inhibitor HCQ, the anti-cancer effect of daphnetin on ovarian cancer cells was enhanced. Daphnetin-induced autophagy and apoptosis may depend on the AMPK/Akt/mTOR pathway in ovarian cancer cells (Lei et al., 2013). Furthermore, daphnetin significantly elevated the level of AMPK in A2780 cells, yet the expression levels of p-Akt and p-mTOR were downregulated. APMK inhibitor (Compound C) reversed the expression of p-Akt and p-mTOR in A2780 cells treated with daphnetin and synergically enhanced daphnetin-induced antitumor effects. Briefly, the Akt/mTOR pathway is involved in Daphnetin-induced protective autophagy and apoptosis (Fan et al., 2021b).

Collectively, daphnetin modulates immune reaction and autophagy, as well as exerts anti-inflammation and anti-cancer effects via the PI3K/AKT pathway.

### 2.4 Effects of daphnetin on JAK2/STAT3 signaling

The JAK2/STAT3 pathway has become a crucial regulator in the initiation and progression of inflammatory and immune responses across a wide range of pathological conditions, thereby exerting significant influence in the pathogenesis of various diseases (Chen et al., 2023; Ott et al., 2023). Janus kinase 2 (JAK2) belongs to the JAK family. These kinases are implicated in immune system regulation, immunocyte differentiation and proliferation, and pro-inflammatory response (Agashe et al., 2022). As a member of the signal transducer and activator of the transcription family, STAT3 acts as a transcription factor, controlling cell cycle progression and apoptotic mechanisms. In addition, STAT3 is also associated with autoimmune and inflammatory diseases (Dong et al., 2021). The JAK2/STAT3 pathway has attracted considerable interest for its distinctive impact on inflammation and lung injury (Figure 4D) (Montero et al., 2021; Liu D. et al., 2022).

Daphnetin has been demonstrated to exert gastrointestinal protective effects, ameliorating the severity of colitis and attenuating the damage to the intestinal structure in DSS-induced ulcerative colitis mice (He et al., 2023). In addition, daphnetin regulated the expression of apoptosis-related proteins *in vivo* at 16 mg/kg for six consecutive days. Daphnetin treatment significantly decreased the level of pro-apoptotic proteins Bax and cleaved caspase 3, while enhanced the anti-apoptotic protein (BCL-2) expression compared with the control group. Daphnetin substantially suppressed the activity and the levels of inflammatory cytokines, including MDA and SOD, conferring anti-inflammatory effects. Likewise, *in vitro* assays verified the cytoprotective effects of daphnetin on Caco-2 cells from LPS-stimulated viability impairment, apoptosis, oxidative stress, and inflammation. Furthermore, daphnetin suppressed the activity of JAK2/STAT3 signaling in LPS-induced Caco-2 cells in a REG3A-dependent manner. Meanwhile, JAK2/STAT signaling inhibition synergized with daphnetin in LPS-stimulated Caco-2 cells. Hence, daphnetin inhibited the UC progression primarily through REG3A-mediated JAK2/STAT3 signaling (He et al., 2023).

By suppressing the JAK2/STAT3 pathway, daphnetin is verified to ameliorate acute lung injury in mice with severe acute pancreatitis (Yang et al., 2021). In the L-arginine-induced SAP-associated acute lung injury model, daphnetin at 2–4 mg/kg significantly reduced IL-6 and TNFα concentrations in both serum and lung tissues, serum amylase and myeloperoxidase activities, and macrophage and neutrophil infiltration and cell apoptosis in the lung tissue; finally alleviating SAP-induced pancreatic and lung tissue damage. Notably, immunohistochemical staining assays suggested that daphnetin pretreatment attenuated the levels of p-JAK2 and p-STAT3, which were comparably increased in the SAP group (Yang et al., 2021). These results are consistent with a previous study that found daphnetin reduces endotoxin lethality and improves LPS-induced acute lung injury in mice via suppressing JAK/STATs activation and ROS production (Shen et al., 2017). Moreover, cell viability was not influenced notably during the daphnetin treatment.

In conclusion, these results showed that inhibiting the JAK2/STAT3 pathway is the essential mechanism of daphnetin to mediate antioxidant activity and anti-inflammatory properties.

### 2.5 Effects of daphnetin on Wnt/GSK-3β/β-catenin signaling

As a highly conserved signaling pathway, the Wnt/β-catenin signaling pathway is pivotal in regulating fundamental physiological and pathological processes, including cell proliferation, survival, differentiation, and migration (Liu J. et al., 2022; Yao et al., 2023). The activation of the canonical Wnt pathway relies on the cooperation between Wnt glycoproteins and several transmembrane receptors (Ma et al., 2023). However, the regulation of β-catenin is influenced by GSK3β, as GSK3β is the upstream molecule of β-catenin. Nonphosphorylated GSK3β can cause the phosphorylation and degradation of β-catenin in the cytoplasm. When GSK3β is inhibited, the phosphorylation of β-catenin will be blocked and cannot be degraded (Figure 4E) (Xia et al., 2019; Cheng et al., 2020).

In a current dexamethasone-induced osteoporosis model, dexamethasone remarkably affected the histological changes, femoral bone mineral content, and femoral microstructure parameters of experimental rats, finally causing osteoporosis (Wang et al., 2020a). However, daphnetin treatment improved bone mineral content and microstructure parameters at 1 and 4 mg/kg, restoring the levels of bone turnover markers (Wang et al., 2020a). Moreover, the Wnt/GSK-3β/β-catenin signaling pathway was stimulated when daphnetin was added, which indicated that daphnetin performed osteoprotective effects via Wnt/GSK-3β/β-catenin signaling pathway. Further study verified this mechanism via XAV939, which inhibits the Wnt/GSK-3β/β-catenin signaling pathway. Once the small-molecule inhibitor XAV939 was added, the transduction of Wnt/GSK-3 β/β-catenin pathway was blocked, which abolished the effect of daphnetin on the differentiation and mineralization of MC3T3-E1 cells, indicating that daphnetin specifically exerted its effects against GIOP via Wnt/GSK-3β/β-catenin pathway (Stakheev et al., 2019; Wang et al., 2020a).

However, daphnetin (25 and 50 mg/kg) is reported to exert antitumor effects by inhibiting the Wnt/β-catenin signaling pathway in hepatocellular carcinoma xenograft models (Liu C. et al., 2022). The roles of daphnetin in apoptosis and G1 phase arrest of hepatocellular carcinoma cells were potently neutralized by activation of the Wnt/β-catenin signaling with SKL2001 treatment, which is an agonist of the Wnt/β-catenin signaling pathway (Liu C. et al., 2022).

Consequently, the effects of daphnetin on Wnt/GSK-3β/β-catenin signaling pathway may vary from the microenvironment of targeting cells; however, daphnetin governs the maintenance of physiological homeostasis by modulating this pathway.

### 2.6 Effects of daphnetin on TGF-β1/Smad2/3 signaling

Known for its pivotal effects on the progression of organic fibrosis, TGF-β1 signaling is closely related to immune response, inflammation, and matrix synthesis (Su et al., 2020; Saadat et al., 2021; Liu Z. et al., 2022). In addition, TGF-β1 can stimulate the phosphorylation of the pro-fibrotic transcription factors Smad2 and Smad3, further driving the expression of TGF-β-sensitive and pro-fibrotic genes (Figure 4F) (Hu et al., 2020).

Currently, a study by Lee et al. explored the effects of daphnetin on transverse aortic constriction (TAC)-induced cardiac hypertrophy and myocardial fibrosis in mice at 10 and 20 mg/kg and angiotensin II (Ang II)–induced hypertrophy in H9c2 cardiomyoblasts at 10 and 20 μg/mL (Syed et al., 2022). The results showed that daphnetin reduced cardiac remodeling by modulating the TGF-β1/Smad2/3 signaling pathway. In addition, daphnetin decreased ECM overproduction, cardiac fibrotic event, and myofibroblast alterations by inhibiting TGF-β1/Smad2/3 signaling proteins, indicating that daphnetin effectively protected against cardiac hypertrophy and fibrosis.

Therefore, daphnetin may have potential therapeutic benefits for cardiac diseases involving heart enlargement and scarring.

### 2.7 Effects of daphnetin on autophagy signaling

Triggered by stress or starvation, autophagy evolves as an intracellular conserved catabolic process mediated by lysosome sustains to degrade cellular components (Yamamoto et al., 2023). During this process, targeting proteins and aged or damaged organelles sequestered in double-membrane vesicles are called autophagosomes, which ultimately fuse to lysosomes, leading to the degradation of the sequestered components (Behrends et al., 2010). The stimulation of autophagy is usually beneficial in disease, as it helps to remove toxic proteins and cells. However, autophagy can serve both tumor-suppressive and tumor-promoting roles, which depend on the tumor stage, biology, and the microenvironment in cancer (Debnath et al., 2023). Therefore, autophagy is a complex and dynamic mechanism that interacts with other cellular pathways in tumorigenesis.

As described above, daphnetin at 10 mg/kg performed antitumor effects in the ovarian cancer A2780 xenograft model (Fan et al., 2021b). Meanwhile, daphnetin also induced autophagy due to the accumulation of LC3-II and endogenous LC3, which was verified as cytoprotective autophagy in ovarian cancer. Because an autophagy inhibitor further enhanced the antitumor efficacy of daphnetin, indicating intricate roles of daphnetin in anti-ovarian cancer effects (Zhang et al., 2019).

By inducing an autophagic response, daphnetin prevents methicillin-resistant *Staphylococcus aureus* and attenuates inflammation (Zhang et al., 2019). A study indicated that daphnetin enhanced microphage bactericidal activity and suppressed inflammatory responses via mTOR-dependent autophagic pathway in C57BL/6 mice. However, once a putative autophagy inhibitor, Bafilomycin A1, was added, the autophagy pathway was blocked, and DAPH-elicited repression of the inflammatory response as well as macrophage antibacterial capability, was abolished (Zhang et al., 2019).

Thus, daphnetin exerts anti-cancer and anti-infection effects by modulating autophagy signaling.

## 3 Effects of daphnetin on NLRP3 inflammasome

Defined as an inflammasome for its ability to respond to DAMPs or PAMPs, NLRP3 Belongs to the nucleotide-binding domain (NBD)- and leucine-rich repeat (LRR)-containing protein (NLR) family, containing a caspase-recruitment domain (ASC) and Caspase-1 (Fu and Wu, 2023). NLRP3 inflammasome functions as a cytosolic signaling complex to mediate the activation of potent inflammation, particularly responding to aging, physical inactivity, over-nutrition, or environmental factors (Mangan et al., 2018).

In the LPS/GalN induced ALF mice model described above, the levels of NLRP3 inflammasome, as well as its downstream inflammatory proteins ASC, Cleaved-caspase-1 (p20), and Mature-IL-1β (p17), were evidently elevated (Lv et al., 2018). As western blotting analysis showed, LPS/GalN activated NLRP3 and related inflammatory proteins were inhibited when treated with daphnetin at 20, 40, and 80 mg/kg (Lv et al., 2018). Therefore, the inflammatory suppression effects of daphnetin are partly attributed to inhibiting NLRP3 inflammasome activation. In addition, daphnetin inhibited the corneal inflammation and neovascularization induced by alkali burn *in vitro* and *vivo*. Moreover, further research showed that alkali burn-induced NLRP3 inflammasome activation was significantly blocked when daphnetin was added, attenuating inflammation and improving wound healing and corneal clarity (Lv et al., 2018; Shimizu et al., 2019).

As mentioned above, the NLRP3 inflammasome exerted a substantial influence on ovarian aging, and high TXNIP protein expression indicates oxidative damage to cells. Daphnetin treatment significantly decreased NLRP3 protein expression compared to the control group, confirming that daphnetin significantly rescued premature ovarian failure (Shimizu et al., 2019).

## 4 Effects of daphnetin on inflammatory factors

As essential mediators, inflammatory factors play critically modificative roles in immune responses (Wang et al., 2022). It is believed that pro-inflammatory factors, including interleukin-1 (IL-1), IL-6, and tumor necrosis factor (TNFα), are dependent on the type I cytokine receptors. While anti-inflammatory factors mainly consist of IL-4 and IL-10 (Turner et al., 2014). Dysregulation in inflammatory factors may cause immune aberrances, hypercoagulability, and reproductive disorders. Intriguingly, daphnetin seems to regulate inflammatory responses by affecting levels of inflammatory factors (Figure 4G).

In the neuropathic pain rats, daphnetin was demonstrated to suppress the expression of pro-inflammatory factors IL-1β, IL-6, and TNF-α, exerting neuroprotective effects. Meanwhile, daphnetin suppressed the activation of microglia, astrocytes, and neurons, thus reducing the nociceptive sensitization in neuropathic pain rats (Zhang et al., 2023). In addition, in an experimental autoimmune encephalomyelitis (EAE) mice model, daphnetin (2 and 8 mg/kg) treatment significantly decreased lymphocyte infiltration and demyelination, which was attributed to reduction in pro-inflammatory factors, including TNF-α and IL-17 and an increase in anti-inflammatory factors, such as IL-4 and IL-10 (Soltanmohammadi et al., 2022). Evidence showed that daphnetin ameliorated the progress of hepatocellular carcinoma by reducing inflammation (Li et al., 2022). Daphnetin(10, 20, and 30 mg/kg) potently suppressed oxidant and inflammatory reactions by reducing the secretion of inflammatory factors TNF-α, IL-1β, and IL-6, ultimately leading to growth cease of hepatic cancer (Kumar et al., 2017; Li et al., 2022).

In fact, these inflammatory factors are secreted by T helper (Th) cells, which can be divided into Th1/Th2/Th17 and regulatory T cells (Tregs) according to specialized functions and patterns of cytokine secretion (Yin et al., 2021). Therefore, the regulatory effects of daphnetin on Th cells have increasingly received widespread attention. By regulating Th17 cells, daphnetin occurred to inhibit immune responses and exert protective effects in a CIA model. Moreover, the level of Th1/Th2 type inflammatory factors was also reduced after daphnetin treatment at 1 and 4 mg/kg (Tu et al., 2012). By modulating both Th17 differentiation and the TGF-β signaling pathway, daphnetin is expected to be a drug candidate for the treatment of idiopathic pulmonary fibrosis, a chronic and refractory interstitial lung disease (Park et al., 2023). As described above, daphnetin at 8 mg/kg profoundly repressed Th1 and Th17 responses, inhibited the secretion of inflammatory factors, and alleviated the clinical symptoms of EAE mice (Wang et al., 2016). Another previous study demonstrated that daphnetin (1 and 4 mg/kg) improved the clinical symptoms and pathological changes in arthritis joints and the beneficial effects associated with restoring the balance of Th cells, including enhancement of Tregs responses and inhibition of Th1/Th2/Th17 cells (Yao et al., 2011). Similarly, in patients with unexplained recurrent pregnancy loss, daphnetin (20 and 40 μg/mL) may exert a regulatory effect on the balance of Th17 and Tregs via decreasing IL-2 and increasing TGF-β1 and IL-6 levels (Zhang Z. et al., 2020). Collectively, daphnetin effectively modulates Th cells and related inflammatory factors.

## 5 Conclusion and perspective

In this review, we provide a novel perspective of the essential molecular effects to elucidate the mechanisms of daphnetin’s sophisticated pharmacological activities. Notably, in almost all research mentioned above, the pharmacological activities of daphnetin are highly likely to increase with its concentration. Furthermore, based on the literature reviewed, it has been reported that daphnetin exhibited a remarkable pharmacological profile. On the one hand, daphnetin can exert diverse molecular effects and pharmacological activities via various signaling pathways, NLRP3 inflammasome, inflammatory cells, and cytokines (Table 1); on the other hand, the specific pharmacological effects of daphnetin within a given signaling pathway can be variable, which depends on the physiological or pathological context present (Javed et al., 2022). Thus, the pharmacological activities of daphnetin may vary with different physiological and pathological contexts, which is due to its distinct interactions with various cell types and the different activation stages of signaling pathways. The diversity of interactions is crucial for understanding and determining the pharmacological effects of daphnetin.

As a natural product, daphnetin has been recognized as an inhibitor of protein kinase, which can partially elucidate the mechanisms underlying the functional diversity of daphnetin. Of coumarin and its derivatives, including daphnetin, esculin, 2-OH-coumarin, 4-OH-coumarin and 7-OH-coumarin, only daphnetin was found to inhibit protein kinases potently. Specifically, daphnetin was verified to inhibit tyrosine-specific protein kinase EGFR (IC_50_ = 7.67 µM) and serine/threonine kinases PKA (IC_50_ = 9.33 µM) and PKC (IC_50_ = 25.01 µM) *in vitro* (Yang et al., 1999). Mechanically, the inhibition of EGF receptor tyrosine kinase by daphnetin was competitive with respect to ATP and non-competitive with respect to the peptide substrate. Moreover, the hydroxylation at the C8 position is likely essential for daphnetin to function as a protein kinase inhibitor when compared to coumarin and its derivatives (Yang et al., 1999).

In addition, the pharmacological activities of daphnetin rely on its effects on various signaling pathways, inflammasomes, inflammatory cells, and cytokines mentioned above. When in the distinct physiological and pathological context, the associated signaling cascades and molecular entities undergo different degrees of dysregulation and disorder. Daphnetin, through its regulatory influence on the equilibrium of these pathways, manifests its therapeutic repertoire, including anti-inflammation, anti-cancer, anti-autoimmune diseases, antibacterial, organic protection, and neuroprotection properties in cell and animal experimental models described above.

Given these underlying mechanisms reviewed, daphnetin is likely to exert a more remarkable pharmacological profile in future research. Hence, we expect daphnetin to be a potential drug candidate for several aberrant disorders, including inflammation-associated diseases, organic injury, cancers, and multidrug-resistant infections. Inspiringly, a clinical trial in which the therapeutic effects of daphnetin on colitis verified its ability to promote the healing of the intestinal mucosa of UC patients and effectively improve the patient’s condition and quality of life (Hu et al., 2021). Moreover, the toxicology studies of daphnetin suggest no morality and other known toxicities.

Still, it is particularly vital to verify the safe dosage range of daphnetin, given its pharmacological activities and pharmacokinetics. Thus, relevant pre-clinical and clinical trials are required for daphnetin’s toxicity assessment and therapeutic application. In addition, the functional effects of daphnetin hinge upon the intricate and interconnected interplay of various mechanisms working in tandem; urgently, there is a lack of detailed mechanisms of daphnetin in epigenetic and metabolic research. Thus, further research is warranted to comprehensively investigate the diverse bioactivities and underlying mechanisms of daphnetin and its derivatives. Additionally, novel combination therapy, including daphnetin and other drugs, needs to be further and extensively investigated.
